# Surface Modification of Anisotropic Dielectric Elastomer Actuators with Uni- and Bi-axially Wrinkled Carbon Electrodes for Wettability Control

**DOI:** 10.1038/s41598-017-06274-0

**Published:** 2017-07-20

**Authors:** Kiwoo Jun, Donggyu Kim, Seunghwa Ryu, Il-Kwon Oh

**Affiliations:** 1 Creative Research Initiative Center for Functionally Antagonistic Nano-Engineering, Department of Mechanical Engineering, Korea Advanced Institute of Science and Technology (KAIST), 291 Daehak-ro, Yuseong-gu, Daejeon 34141 Republic of Korea; 2 Department of Mechanical Engineering, Korea Advanced Institute of Science and Technology (KAIST), 291 Daehak-ro, Yuseong-gu, Daejeon 34141 Republic of Korea

## Abstract

Interest in soft actuators for next-generation electronic devices, such as wearable electronics, haptic feedback systems, rollable flexible displays, and soft robotics, is rapidly growing. However, for more practical applications in diverse electronic devices, soft actuators require multiple functionalities including anisotropic actuation in three-dimensional space, active tactile feedback, and controllable wettability. Herein, we report anisotropic dielectric elastomer actuators with uni- and bi-axially wrinkled carbon black electrodes that are formed through pre-streching and relaxation processes. The wrinkled dielectric elastomer actuator (WDEA) that shows directional actuation under electric fields is used to control the anisotropic wettability. The morphology changes of the electrode surfaces under various electric stimuli are investigated by measuring the contact angles of water droplets, and the results show that the controllable wettability has a broad range from 141° to 161° along the wrinkle direction. The present study successfully demonstrates that the WDEA under electrically controlled inputs can be used to modulate the uni- or bi-axially wrinkled electrode surfaces with continous roughness levels. The controllable wrinkled structures can play an important role in creating adaptable water repellency and tunable anisotropic wettability.

## Introduction

The growing interest in human-friendly actuator devices for industrial applications, and in wearable consumer products to enhance personal convenience, has led to the rapid development of soft actuators^1–5^. Soft actuators are devices that exhibit stretchable, flexible, and deformable behaviors with the application of an electrical, chemical, or thermal stimulus^6^. In particular, electroactive polymer actuators (EAPs), which include ionic polymer metal composites (IPMC), dielectric elastomers, conducting polymers, and polymer gels, have been intensively studied, because of their great potential for use in soft actuating systems^7^. Among the various EAPs, dielectric elastomer actuators (DEAs) constitute a class of macromolecules that experience an in-plane areal change in response to a generated electrostatic force. DEAs are relatively inexpensive, lightweight, and easily produced, and have high dimensional actuation strain (from 50–380%) with moderate blocking stresses. They also have a wide range of energy densities (0.01–3 MJm^−3^), and high electromechanical coupling efficiencies (~90%) over a broad range of electrostatic fields (70–400 Vμm^−1^)^8–11^.

The conventional DEA technologies have been applied to haptic devices to generate tactile feedback^12^ in response to touch by fingers, and a tubular actuating system with a cylindrical configuration^13^ to provide longitudinal motion. Practical application systems require more complex actuation capabilities for multiple functionalities rather than simple changes in dimension and shape. Recently, DEAs have been studied to enhance the actuation performance, or to provide new functionalities, by incorporating corrugated structures on the surface of the elastomer film^2, 14–17^. For example, anisotropic dielectric elastomer actuator with tunability has been suggested by Ghosh and Spontak^18, 19^. They reported that the dielectric elastomer actuator generates high anisotropy over 2.0 and a relatively large directional strain with sufficient fiber concentration (5.2 vol%), when the dielectric elastomers are uni-directionally constrained by the aligned PU-fiber. They also showed that combining the DEAs with an arranged carbon nanotube (CNT) sheet provided vastly improved directional strain response at a relatively low electric field (100 Vμm^−1^).

In addition to the anisotropic behavior of the DEAs, a wrinkled structure formed on the elastomer surface has been of interest in various engineering fields. Wrinkles are unique surface patterns formed on the surface of soft materials as a result of mechanical instability. They are generated when a material system is under both equilibrium and non-equilibrium states. Some of these instabilities are elastic in nature, and as such they can disappear or reappear with the control of an external force^20^. Previously, such elastic instabilities in soft materials, including liquids, elastomers, elastoplastic polymers, and hydrogels, have often been perceived as failure mechanisms. In recent times, these instabilities have been exploited in a wide range of application fields related to surface topography and its dynamic tuning, such as assembling complex patterns^21, 22^, fabricating novel electronic devices^23, 24^, and tuning diffraction gratings^25^. In particular, Stafford *et al*. have investigated liquid wetting on wrinkled solid materials to control surface wetting and adhesion^26^. They provided new insights into the interpretation of wetting properties and related liquid contact angle measurements on anisotropic micro-wrinkled surfaces. To the best of our knowledge, even though wrinkles are mechanically tunable and variable under an external stimulus^26, 27^, anisotropically wrinkled dielectric elastomeric actuators, which can be used to tune their macro- and microscopic shape under electrical stimulus, have not until now been applied to the directional control of hydrophobic surface morphologies.

Herein, we report uni- or bi-axially wrinkled dielectric elastomer actuators (designated WDEAs) that have mechanically buckled electrodes on elastomer surfaces, and that can provide directional actuation and anisotropic wettability. The surface characteristics of mechanically generated wrinkles were analyzed by AFM and FE-SEM, and the contact angle of water droplets. Furthermore, calculations based on wetting theory were also compared with the experimental results. The wrinkled dielectric elastomer actuator was electrically actuated to confirm the modification of its surface morphology with various input voltages. The morphological changes of the wrinkles on the elastomer film were verified by measuring the contact angle of water droplets under various surface conditions, resulting in a broad range of tunable wettability states from 141° to 161° along the wrinkle direction. Those findings supported that the various roughness levels in the controllable anisotropic micropatterns played an important role in tunable water repellence and the anisotropic behaviors of water droplets.

## Results and Discussion

**Figure 1:**
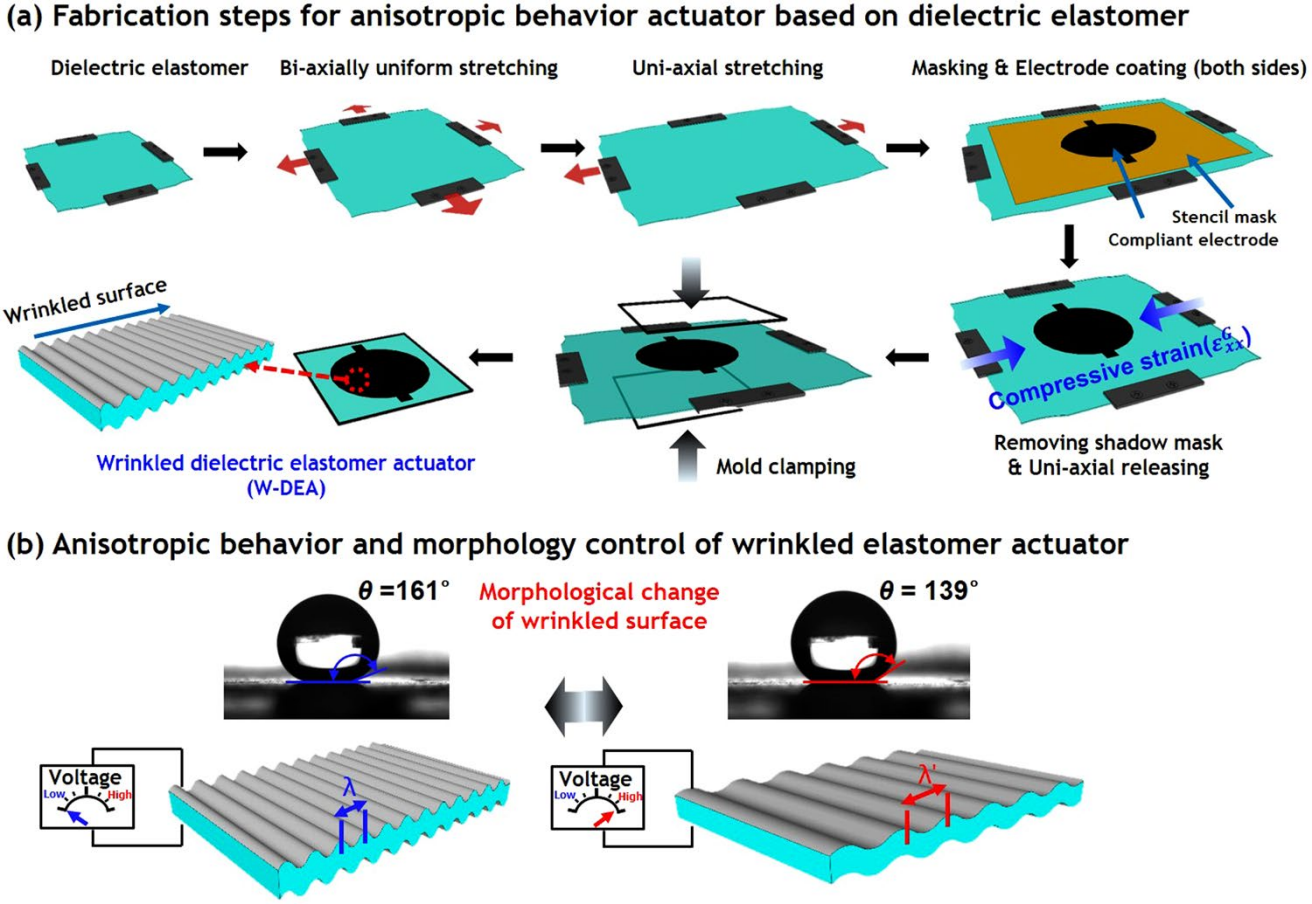
Schematics of the wrinkled dielectric elastomer actuator: the (**a**) fabrication processes, and (**b**) operating principle of the uni-axially wrinkled anisotropic elastomer actuator.

Figure 1 shows the fabrication process of the uni- and bi-axially wrinkled surfaces and the operating principle of the WDEA. To prepare the WDEA, an acrylic elastomer film (VHB 4905) was bi-axially stretched to twice its original dimension simultaneously (that is, pre-strained by 100%), and maintained in the pre-stretched state. It is well known that stretching the elastomer film results in improved actuation performance with respect to its breakdown strength, actuation strain and energy efficiency.

To produce the uni-axially wrinkled pattern on the elastomer surface, the elastomer was stretched by 100% more in the x-direction (that is, pre-strained by 200% in the x-direction) while the y-axis was maintained in the 100% pre-strained state. Carbon black powders were then deposited with a brush on both the upper and lower surfaces of the pre-strained elastomer film. Figure 1a shows that thereafter, the x-directional pre-strains of the carbon black-coated elastomer were relaxed back to the initial value of the pre-stretched state (100%). During the relaxation process of the elastomeric film in the x-direction, the lateral dimensions of the active area (the carbon black-coated area) were reduced macroscopically by the same ratio as those of the film. At the microscopic scale, when the substrate was relaxed along the x-axis, the carbon black electrodes on the elastomer film developed wrinkles and a buckled morphology^27^.

When an external electrical voltage is applied to compliant electrodes, the Coulomb force between the opposite charges of both electrodes generates an electrostatic force or pressure on elastic film, causing it to squeeze in the thickness direction, while expanding the active area of the electrodes along the in-plane directions. The electrostatic force, also termed the Maxwell stress, *p*, can be expressed as $p={\varepsilon }_{0}{\varepsilon }_{r}{(V/z)}^{2}={\varepsilon }_{0}{\varepsilon }_{r}{E}^{2}$ ^28^. Here, *ε*_0_ is the vacuum permittivity, *ε*_*r*_ is the relative permittivity of the elastomer, *z* is the thickness of the elastic film, and *V* is the applied voltage. Under this squeezing force, conventional DEAs, which are equi-biaxially pre-stretched in the x- and y-axis directions, show an equal actuation strain response in the active area, before dielectric breakdown of the DEAs occurs^29, 30^.

The working principle of the WDEAs is similar to that of conventional DEAs. The actuator also expands its active area along the in-plane direction due to the attractive force between the two electrodes, until the applied voltage on the electrodes reaches a critical value termed the breakdown voltage. However, in the case of the WDEAs, there is an anisotropic difference in the actuation behavior, which can be observed in the actuation displacements perpendicular, and parallel, to the wrinkle directions. Because the sinusoidal pattern of the surface has a geometrically longer line-length in the wrinkle direction as opposed to the non-wrinkle direction, this structurally asymmetric system produces highly stretchable and anisotropic actuation behavior in the uni-axially wrinkled elastomer actuator. This anisotropic behavior ultimately results in anisotropic wetting controllability, because the wrinkled surface morphology of the WDEAs can be varied as the function of an activating voltage.

**Figure 2:**
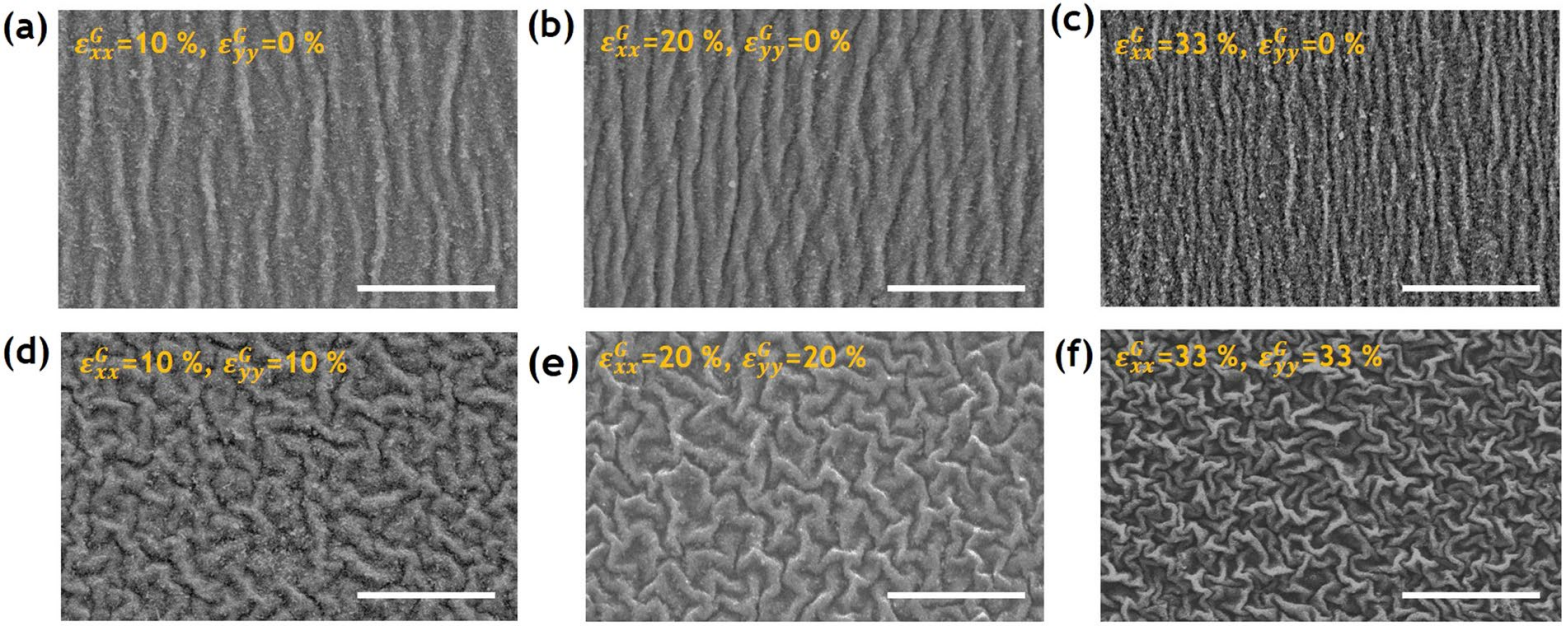
SEM imagery of the wrinkled electrode showing one- and two-directional ripple patterns due to the buckling of the carbon electrode: uni-axially wrinkled surfaces produced by compressive strains of (**a**) 10, (**b**) 20, and (**c**) 33%; bi-axially wrinkled surfaces produced by compressive strains of (**d**) 10, (**e**) 20, and (**f**) 33%, in both the x- and y- directions (Scale bar = 20 μm).

Figure 2 shows scanning electron microscope (SEM) images of the formation and evolution of the uni-axial wrinkles for various compressive strain values. According to previous studies, the process of wrinkle formation patterns can be explained by considering the total energy, which is the sum of the stretching energy of the soft elastomer, and the bending energy of the hard surface electrode^31^. When an external compressive stress is applied to a thin film electrode in the plane direction, elastic instability occurs beyond a critical stress, causing the flat carbon electrode to be buckled and wrinkled and resulting in out-of-plane deformations. If the thin film electrode is coupled to soft elastic materials, such as PDMS, eco-flex, or VHB film, a periodic pattern of buckles, generally referred to as wrinkles, appears on the surface of the soft materials^32^. The wavelength (*λ*) and amplitude (*A*) of the generated wrinkle is decided as a function of the thickness and mechanical properties of the electrode.

In this study, we focused on the formation of wrinkled carbon electrodes under uni-axial and bi-axial compression after pre-stretching the elastomer film. As the pre-stretched elastomer film is gradually relaxed along one direction, the apparent length of the film reduces from *L*_*0*_ in the initially stretched state to *L* in the final relaxed state. We define the macroscopic compressive strain of carbon black electrodes in the elastomer film along the relaxed direction as ${\varepsilon }^{G}=({L}_{0}-L)/{L}_{0}$. The compressive strain in an electrode can be calculated as *ε* ^*G*^ *=* (*ε*_*pre*_ − ε)/(*ε*_*pre*_ + *1*), where *ε*_*pre*_ is the pre-strain of the substrate, and *ε* is the tensile strain in the substrate in the final state. When the compressive strains in the carbon black electrode reach the critical value, the wrinkles develop with an initial wavelength, as follows:

1 $${\lambda }_{0}=2\pi t{[\frac{E}{12\Lambda {\mu }_{s}(1-{\nu }^{2})}]}^{\frac{1}{3}}$$

Where, *E*, *t*, *ν*, and *μ*_*s*_ are the Young’s modulus, thickness of the electrode film, Poisson’s ratio of the thin film electrode, and shear modulus of the substrate taken to be a neo-Hookean material, respectively^32, 33^. Also, the parameter (*Λ*) can be expressed as $\Lambda =(1+{(1+{\varepsilon }_{pre})}^{3}/2(1+{\varepsilon }_{pre})$.

In the present case, the wavelength (*λ*_0_) and amplitude (*A*) of these wrinkles were simply adjusted by varying the strain values (*ε* ^*G*^), because the wavelength (*λ*_0_) corresponds to the compressive strain value (*ε* ^*G*^). The wavelength (*λ*_0_) of the wrinkled carbon electrodes on elastomeric film was found to vary from 4 to 2 μm, in accordance with varying the compressive strain *ε* ^*G*^ from 0 to 33%, respectively (Fig. 2a–c). The *λ*_0_ value of the wrinkles was measured by low-magnification SEM. The average distance between five pairs of two consecutive wrinkles was considered. There were differences in *λ* value across the wrinkled surface, because of the non-uniform distribution of the compressive strain. Inhomogeneity in the amplitude of wrinkles also increases as the compressive strain increases^33^.

**Figure 3:**
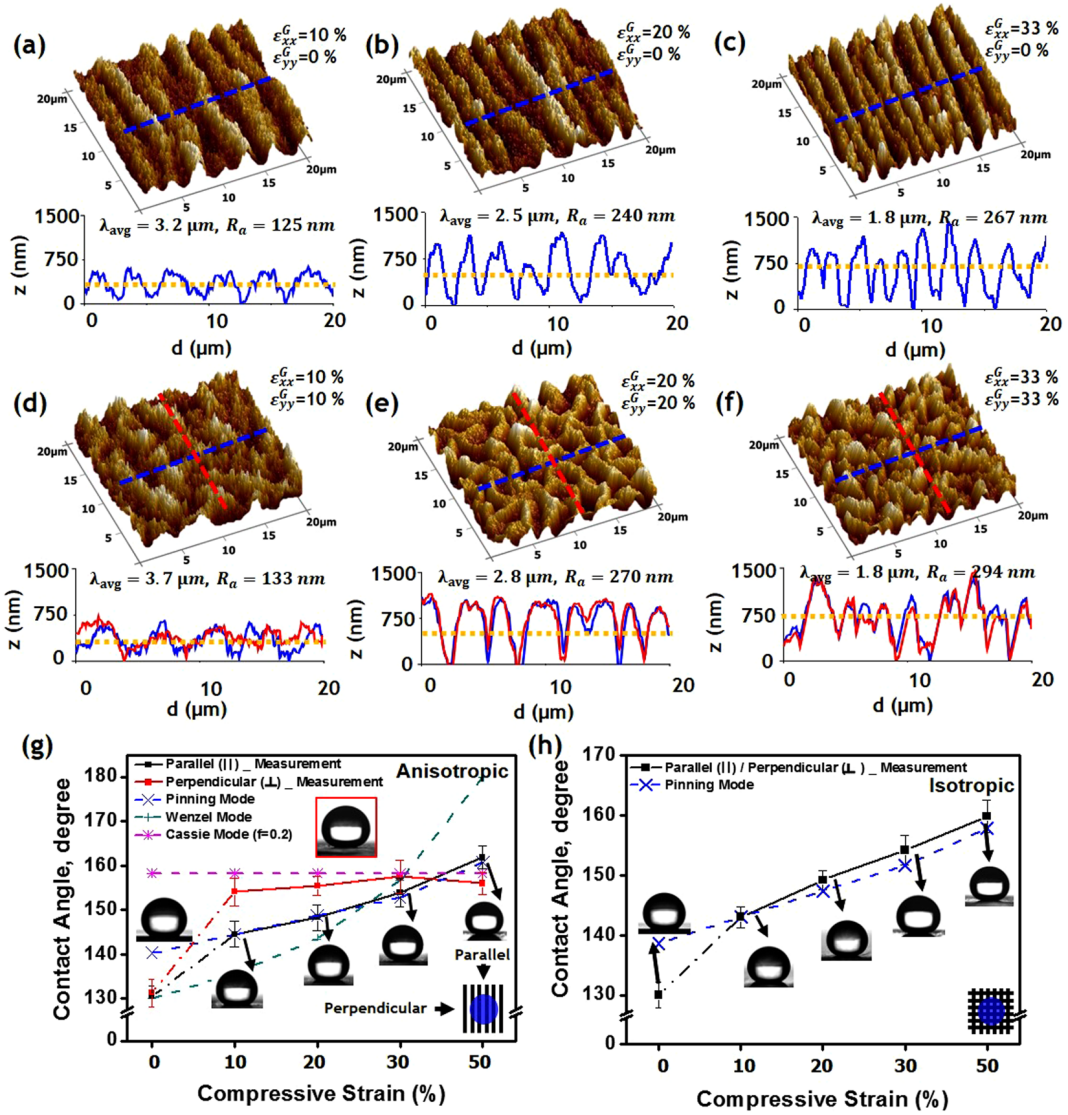
AFM analysis and contact angle of wrinkled pattern with increasing level of surface roughness. The surface morphology shifts from wrinkles having long wavelength and low height (**a**) and (**d**), to a uniformly structured surface with various levels of surface roughness (**b**,**c**) and (**e**, **f**). *R*_*α*_ indicates the average surface roughness, measured on 20 μm ×20 μm regions. On the bottom of each substrate, blue and red lines stand for the profiles of the surface, and the yellow line shows the average amplitude of the wrinkle. (**g**, **h**) static water contact angle as a function of the compressive strain %. The insets of (**g**) show the anisotropic wettability behavior of the water droplet contact angle along the wrinkle direction; the angle in the parallel direction increases with the strain value, while a stable contact angle is maintained in the perpendicular direction.

To investigate how surface roughness changes with the variations of the wrinkle conditions, we measured the contact angle of water droplets with three types of uni- and bi-axial wrinkled surfaces. Along with the experiments, we conducted theoretical calculations of contact angles, by considering the various wetting modes on the wrinkled surfaces. Figure 3 shows atomic force microscopy (AFM) images of the microstructured surfaces with various wavelengths and amplitudes between parallel ridges. It is interesting to take note of the difference in the surface roughness for uni-axially and bi-axially compressive strain states. With uni-axial compression, the roughness value (*R*_*a*_) for uni-directional wrinkles, which is the arithmetic average of the surface height deviations measured from the mean plane within the target area, increased from 125 nm for the first microstructure sample (Fig. 3a) to 267 nm for the roughest surface (Fig. 3c). In comparison, for the bi-axial wrinkle under simultaneous bi-axial compression in the x- and y-directions, *R*_*a*_ varied from 133 to 294 nm. The AFM line profiles (bottom graphs of Fig. 3a–f) of the rough substrates confirmed a homogeneous increase in the roughness of the various samples, where the surface morphology was controlled by bi-axial tension.

The anisotropic wetting of the wrinkled surfaces was investigated by measuring the static water contact angles, both perpendicular (*θ*_⊥_) and parallel (*θ*_*||*_) to the wrinkle direction^34^. Figure 3g–h show that as the surface microstructure roughened, the static contact angles increased, and reached a saturating value. Initially, the non-wrinkled film had an isotropic contact angle of 130° ± 2° with small surface roughness values. At the increased compressive strain values (${{\epsilon }}^{G}=10 \%$), the film had a wavy pattern, with *θ*_⊥_ value of 152° ± 2°, and *θ*_*||*_ value of 144° ± 3°. These anisotropic wetting properties resulted from the directional pinning barrier caused by the wrinkle heights and amplitude. The *θ*_*||*_ increased steadily with compression, and reached 163° ± 2° at a strain of 50%, while *θ*_⊥_ increased quickly, and remained at about 153°; this indicates the transition from isotropic to anisotropic wettability. In contrast, the bi-axially wrinkled surface had a linear variation of isotropic contact angles.

The changes of the contact angle can be explained by considering various wetting modes on the wrinkled surface. The wetting state on the rough hydrophobic surface is determined by the lower free energy state out of the Wenzel mode^35^, (where the grooves are filled with liquid), and the Cassie-Baxter mode^36^, (where the grooves are filled with air). Each of the wetting states predicts contact angles as follows:

2 $$cos\theta ^{\prime} =rcos{\theta }_{0}({\rm{Wenzel}},{\rm{W}}\,{\rm{mode}}),$$

3 $$cos\theta ^{\prime} =fcos{\theta }_{0}+f-1\,(\mathrm{Cassie} \mbox{-} \mathrm{Baxter},{\rm{CB}}\,{\rm{mode}}).$$

In the above expressions, *r* denotes the roughness factor, which indicates the ratio of the true surface area to the projected area, and it can be expressed with compressive strain^37^, as $r=\frac{1}{1-{\epsilon }}$. The other roughness factor, *f*, denotes the fraction of the wetted area from the projected area, *θ*_0_ denotes the Young’s angle^38^ on the flat surface, and $\theta \text{'}$ denotes the contact angle on the rough surface. Of the two wetting modes, the mode with the smaller contact angle has less free energy, and is chosen as the stable state^39^.

It was found that as the compressive strain increased, the measured contact angle of the perpendicular direction (*θ*_⊥_) remained constant; this can be explained by the CB mode with *f* = 0.2. Even though the Wenzel mode is the lower free energy state when the strain is less than 0.3, the wetting state can be determined by the meta-stable CB mode due to the energy barrier between the W and CB modes^39^. On the other hand, *θ*_*||*_ does not match either the W mode or CB mode prediction, and can only be explained by considering pinning of the liquid tip. The liquid tip can be confined within a local free energy minimum, when it is pinned by a high protuberance of the rough surface. The pinning energy barrier is maximized at the highest protuberance. We expect that relatively high amplitude wrinkling occurs at the positions with low bending modulus (or surface defects) under the onset of the initial wrinkling; and those positions would remain as relatively high protuberances, until the compressive strain significantly increased (Supplementary Fig. S4)^37^. If we assume that the baseline width of the liquid droplet is determined by a few high protuberances, and hence reduces in proportion to the compressive strain, the contact angle is predicted to be the blue dot in Fig. 3g, which shows good agreement with the measured contact angles (Supplementary Fig. S4). In the bi-axial case, we adopted the same analysis, and found that the measured contact angles can be explained by considering the pinning state.

**Figure 4:**
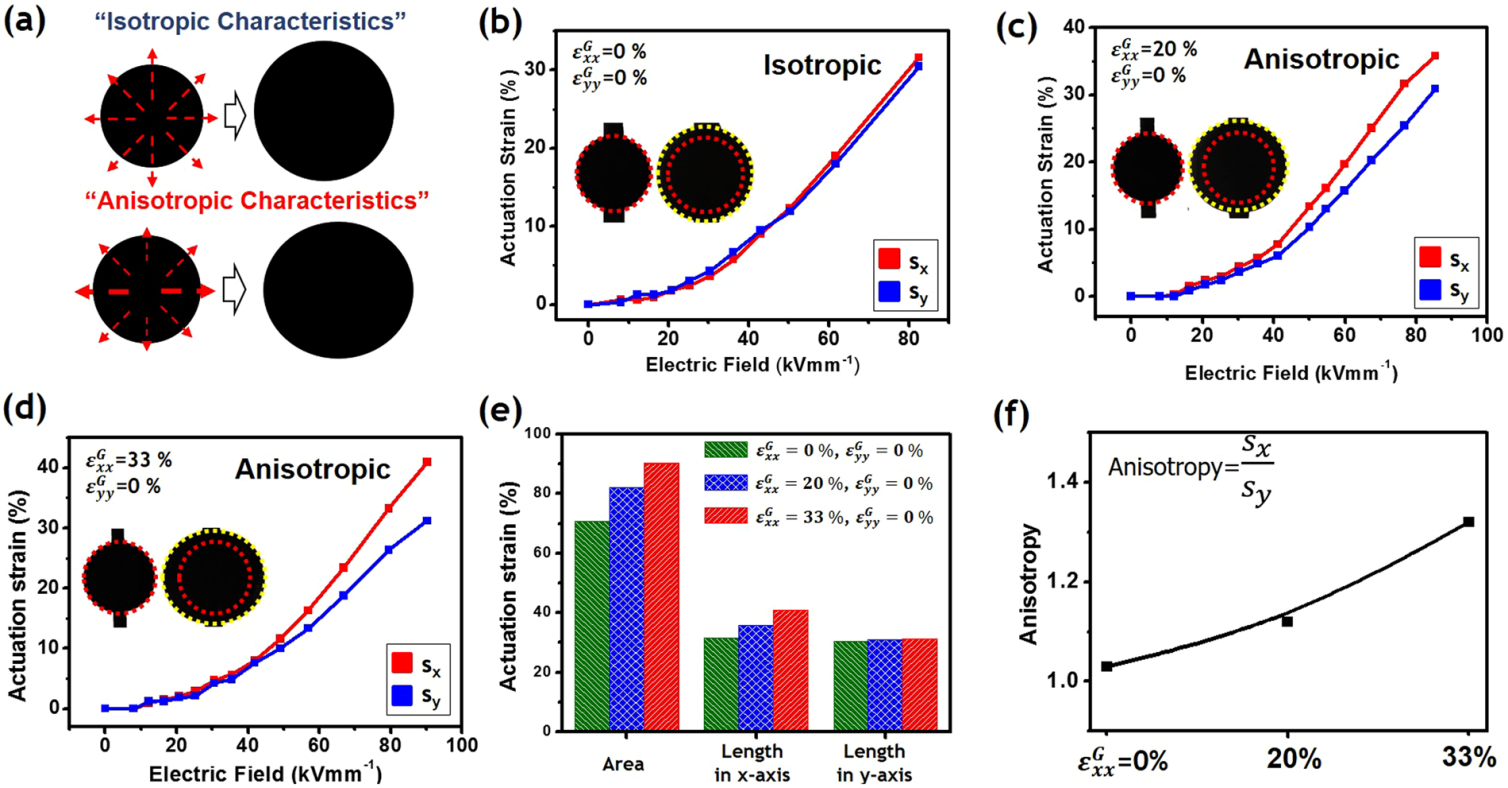
Macroscopic observation of wrinkled elastomer actuators: (**a**) concept of anisotropic actuation, (**b**) equibiaxially-prestretched elastomer actuator, (**c**) wrinkled elastomer actuator with ${\varepsilon }_{xx}^{G}\,$= 20% (**d**) wrinkled elastomer actuator with ${\varepsilon }_{xx}^{G}$ = 33%, (**e**) maximum areal and directional strains with various levels of compressive strain, and (**f**) anisotropy value according to different compressive strains.

The activation of conventional DEAs using a high input-voltage caused the electrodes to expand with a isotropic areal strain. Figure 4 shows that the isotropic and anisotropic behaviors of DEAs can be differently realized by the morphological configuration of carbon electrodes. Linear actuation strains, s_x_ and s_y_ in the x- and y-directions, respectively, were measured from three different types of WDEAs with ${\varepsilon }_{xx}^{G}$= 0, 20, and 33% in the x-direction, and are presented as a function of the electrical field (kVmm^−1^). In Fig. 4b–d, the inset images display the different deformed images of three types of WDEAs. The red dotted circle represents the edge line of an initial active area on the actuator electrode, and the yellow dotted circle represents the edge line of an expanded active area before dielectric breakdown. The axial strains in the x-direction have increased value as ${\varepsilon }_{xx}^{G}$ increases, and the strain values of the other direction have almost the same strain value. Even though they all had the same elastomer pre-stretching ratio, the directional strain of the anisotropic WDEAs can be achieved with the extension of buckled electrodes. In other words, the DEAs with non-wrinkled electrodes had an actuation strain property that was indistinguishable over the whole input voltage range.

The axial strain difference (s_x_ − s_y_) was 0, 5, and 10 for ${\varepsilon }_{xx}^{G}=0,\,20$, and 33%, respectively. For areal actuation strains, the DEAs with plain thin film electrodes showed less actuation strain by about 20% due to the relatively large stiffness of their electrodes, compared with the buckled states (Fig. 4e). Also, DEAs with wrinkled thin film electrodes generally fared better with higher compressive strain. Except the case of ${\varepsilon }_{xx}^{G}={\varepsilon }_{yy}^{G}=0 \%$, s_x_ is greater than s_y_, and as the wavelength of the wrinkle decreases, the anisotropy value increases. Since a purely mechanical response would produce a large strain along the x direction and a relatively small strain in the orthogonal direction (y), this result provides the direct evidence for the existence of a coupled electromechanical interaction between the anisotropic electrical behavior and compressive strain value^18^. Here, an important observation is the stretchability of the actuator electrode along the wrinkle direction. The actuation performance is associated with the morphological pattern, and the wrinkled periodic structures are more stretchable than flat and non-periodic structures in most cases. The periodic ridge patterns as a compliant electrode have an absolute advantage in realizing anisotropic actuation.

To verify the geometric advantages in terms of electrical resistance, the change in morphology of the wrinkled electrode upon uni-directional tensile strain is shown in Supplementary Fig. S2. The optical microscopy images of the wrinkled carbon electrodes at various strain levels in the x-axis shows the transition process of the wavelength and amplitude from 0 to 30% strain value. As the nominal tensile strain *ε*_*x*_ further increases, the amplitude of the generated wrinkles gradually decreases. With further increment of *ε*_*x*_, the sinusoidal wrinkle pattern on the surface subsides, and the amplitude of the ridges dwindles. Once the substrate is fully stretched, the pattern of localized ridges with the wavelength of ~5 μm eventually changes to a soft and flat surface state. Meanwhile, with increments of *ε*_*y*_, the lines of electrode fracture occur in a direction parallel to the applied tensile strain at a relatively low strain level (15%), compared with the level of the x-direction.

To investigate changes in the electrical conductivity of the wrinkled electrodes with morphological changes, the resistance of the wrinkled electrodes was measured as a function of a tensile strain along the x-direction. The normalized resistance values along both the x- and y-directions changed slightly near zero strain. The change in the electrical resistance in the x-direction was initially slower, up to about 20% mechanical strain, without a significant loss of conducting pathways through the wrinkle surface. In the y-direction, beyond 30% strain, the electrodes were in an almost insulating state. For the x-direction strain, the change in the electrical resistance was gradual and more modest. These electrodes resulted in enhanced directional actuation responses that highly correlate with the generated wrinkle direction.

**Figure 5:**
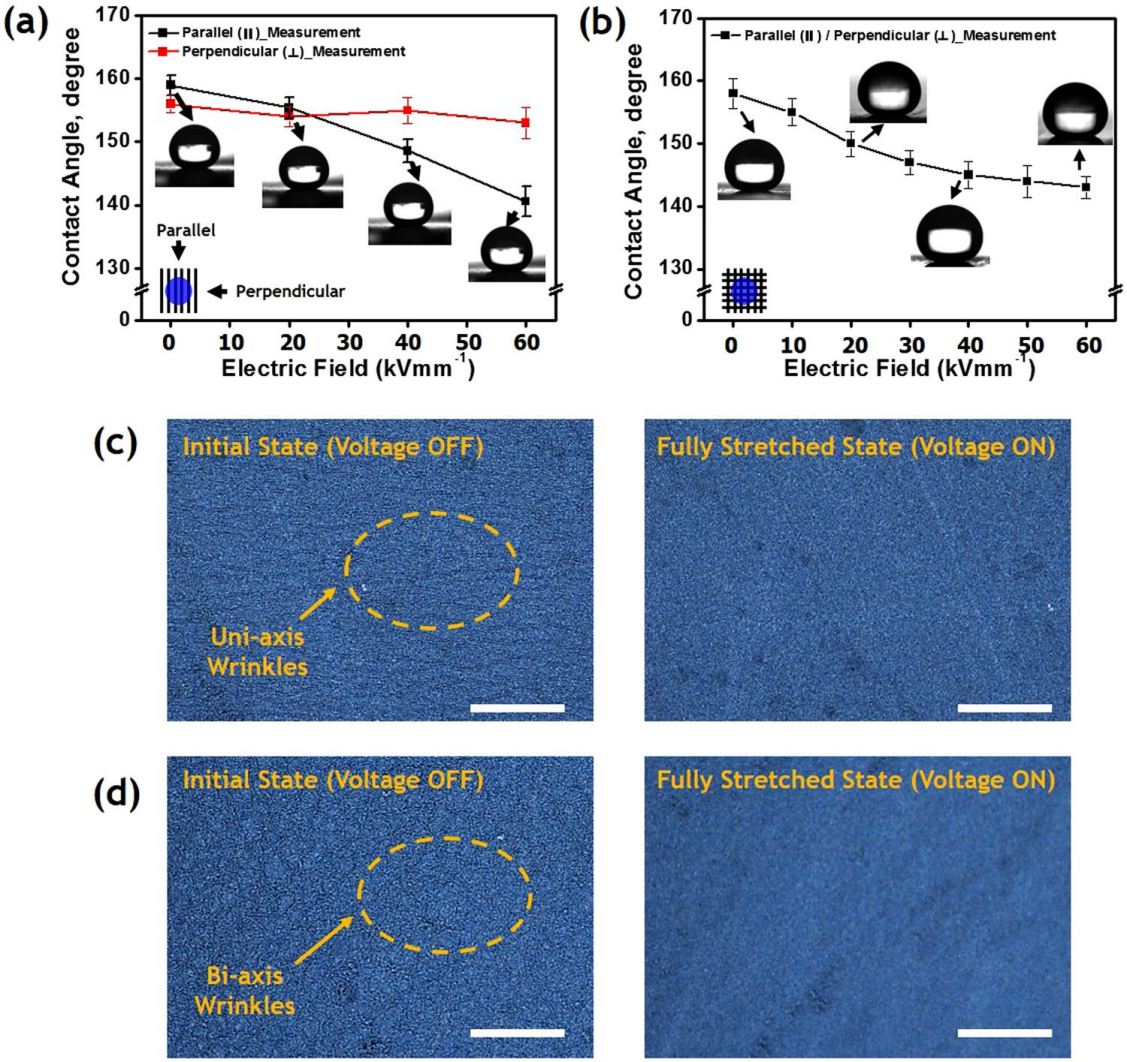
Anisotropic and isotropic wettability control due to surface modification of winkled elastomer actuator: contact angle variation of a water droplet on (**a**) uni-axial, and (**b**) bi-axial wrinkled surface; and microscopic observation of wrinkled surface modification under WDEA actuation: (**c**) uni-axial and (**b**) bi-axial wrinkled surface (Scale bar = 100 μm).

In-plane deformations of the wrinkled dielectric elastomer actuators provide areal expansion, and directionally enhanced actuation behavior of the wrinkled active areas. To confirm the variation in surface roughness when the WDEAs were activated, we measured the contact angle of water droplets on the WDEAs, and observed the microscopic pattern of the wrinkled surface. Figure 5 shows the variation in the contact angle. We employed a microstructured surface having a wavelength of 1.8 μm and a roughness of 267 nm in this experiment. The electric field (*E*) was increased from 0 to 60 kVmm^−1^ to investigate the actuation performances of the uni- and bi-axially wrinkled acutators. In these cases, as the electric field *E* increased, the surface roughness decreased.

Under this condition, the anisotropic wettability of the wrinkled surfaces was investigated with two othogonal contact angles, both perpendicular (*θ*_⊥_) and parallel (*θ*_||_) to the wrinkle direction. Figure 5a shows that as the surface microstructure roughened, the static contact angles decreased, and reached a saturating value. Initially, the uni-axially wrinkled surface has a perpendicular contact angle, *θ*_⊥_, of 154° ± 2° and a parallel contact angle, *θ*_||_, of 159° ± 2° at larger roughness values of the surface. With the increased electric field, the film has a *θ*_⊥_ value of 152° ± 2° and a *θ*_||_ value of 143° ± 3° on the wavy pattern.

These anisotropic wetting properties resulted from the directional pinning barrier caused by the wrinkle heights, amplitude, and surface roughness. The *θ*_||_ value decreased with increasing electrical potential between the compliant electrodes of the wrinkled acutator, while *θ*_⊥_ remained at about 153°, which indicates that the wettability transitioned from isotropic to anisotropic. Otherwise, the bi-axially wrinkled surface simply exhibited a linear variation in water contact angle that was irrespective of the wrinkle direction.

In the experiment with the uni- and bi-axial wrinkled surfaces, the patterns of the parallel and randomly-distributed ridges varied in terms of the amplitude, wavelength, and roughness. Figure 5c–d show that the roughness of the uni-axially wrinkled surface was decreased from 267 to 125 nm. In the case of the bi-axially wrinkled surface, the roughness value changed from 294 to 133 nm. These results indicate that the anisotropic wetting properties could be accurately controlled by the surface modification of WDEA, combined with the wrinkle direction.

## Conclusion

In summary, we report the control of the anistropic wetting utilizing wrinkled dielectric elastomer actuators (WDEAs) that were fabricated with mechanically buckled carbon black electrodes on elastomer surfaces through bi-axial pre-stretching and controlled relaxation processes. The WDEAs under applied electrical fields showed directionally anisotropic actuations and the hydrophobic surfaces of uni-axially or bi-axially wrinkled carbon electrodes were controlled under the electric stimuli, resulting in tunable anisotropic wettability. The contact angles of water droplets on the wrinkled carbon electrodes show a broad range of tunable wettability from 141° to 161° along the wrinkle direction. Theoretical calculations based on wetting theory were also compared with these experimental results. The ridges and vertices in the wrinkled surface were highly deformed and microscopically patterned, which can potentially lead to uni- and bi-axial actuation modes and directional wettability. Furthermore, it will be possible to develop active tactile texture devices with novel tunability and flexibility by using an elastomeric actuation system to control the microscopic pattern of the wrinkled surface.

## Methods

### Preparation of Wrinkled Electrodes on Elastomeric Film

A process of pre-stretching and releasing the elastomer film was used to generate the uni-axial and bi-axial wrinkles on the elastic surface. The dielectric elastomer was stretched to a ratio of *ε*_*pre*_, and then both sides of the elastomer surface were coated with carbon black powder while it remained under the pre-stretched condition (Supplementary Fig. S1). To generate a buckled electrode having various wavelengths and amplitudes, the compressive strain value of *ε* ^*G*^ in the wrinkled electrodes was properly controlled.

### Preparation of Wrinkled Dielectric Elastomer Actuator

An acrylate adhesive film (VHB 4905, 3 M Corp.) was uni- and bi-axially stretched to 100% in the plane direction, and was then fixed on a customized stretching machine (Supplementary Fig. S1). After further stretching the elastomer film, carbon black powders were painted by brush on both sides of the pre-strained film. A laser-scribed stencil mask (made of polyethylene film) with a circular active area of 20 mm in diameter was used to make circular-shaped DEAs. The x-axis length of the stencil mask ranged from 35 to 20 mm with respect to the axial stretch ratio, while the y-axis length of the stencil mask was 20 mm. The carbon particles were easily coated on the sticky surfaces of the elastomer by gentle brushing through the circular masks. After adjustment of the stretching ratio of the film, each specimen was fixed with a rigid frame.

### Microscopic Patterns of Wrinkled Surface on Elastomer Substrates

High-magnification image of the wrinkled surfaces was obtained with field-emission scanning electron microscopy (Hitachi, S-4800) under an accelerating voltage of 10 kV. Low-magnification image of the wrinkled surfaces was obtained with optical microscopy (Nikon, Eclipse LV150N) under multiple levels of tensile strain. The heights and amplitudes of the generated wrinkled surface were checked and confirmed by typical tapping-mode atomic force microscopy (AFM) measurement.

### Macroscopic and Microscopic Observation (Actuation Characterization)

An active area located in the middle of each specimen was fabricated by coating carbon black particles (purchased from Samjung C&G) on the pre-stretched dielectric elastomer. Each specimen was fixed in the rigid test frame. The electrodes of the active area were connected to a high-voltage power supply (Trek Model 610E) using a copper wire. After connecting the electrodes to the supply, the actuation of the WDEAs was driven by a manual input voltage trigger. The real-time in-plane expansion of the active area with increasing input voltage was recorded using a DSLR camera (EOS 650D, Canon). The images were captured by an image processing program, and the captured images were analyzed with ImageJ (Image Analysis Program, National Institutes of Health) to quantify the corresponding electro-actuation strain (s_x_ and s_y_) in the x- and y- directions. Using this experiment set-up, the contact angle of water droplets was measured when the wrinkled surface was modified under the applied electrical fields between the elastomer.

## Electronic supplementary material

Supporting Information
