# Supplementary material for: Surface Modification of Anisotropic Dielectric Elastomer Actuators with Uni- and Bi-axially Wrinkled Carbon Electrodes for Wettability Control
Source: Sci Rep. 2017 Jul 20;7:6091. doi: 10.1038/s41598-017-06274-0 (PMC5519688; doi:10.1038/s41598-017-06274-0)
Supplement: Supplementary file 1 — Supporting Information [file 41598_2017_6274_MOESM1_ESM.pdf]

Supporting Information

Title

Surface Modification of Anisotropic Dielectric Elastomer Actuators with Uni- and Bi-axially Wrinkled Carbon Electrodes for Wettability Control

Kiwoo Jun[1], *Donggyu Kim*[2], *Seunghwa Ryu*[2], *and Il-Kwon Oh*[1,*]

[1] Creative Research Initiative Center for Functionally Antagonistic Nano-Engineering, Department of Mechanical Engineering, Korea Advanced Institute of Science and Technology (KAIST),291 Daehak-ro, Yuseong-gu, Daejeon 34141, Republic of Korea
Kiwoo Jun & Il-Kwon Oh

[2] Department of Mechanical Engineering, Korea Advanced Institute of Science and Technology (KAIST),291 Daehak-ro, Yuseong-gu, Daejeon 34141, Republic of Korea
Donggyu Kim & Seunghwa Ryu

* Corresponding Author: Tel.: +82-42-350-1520, E-mail: ikoh@kaist.ac.kr

Keywords: surface modification, dielectric elastomer actuator; wettability; wrinkle; anisotropy

Supplementary Note 1. Film stretching frame and fabrication process for WDEA

To make the wrinkled elastomer actuator, pre-stretching and releasing processes of the elastomer film were conducted with a customized stretching machine as shown in **Figure S1**. After the film was loaded into the clamps, the film was stretched in the x- and y-directions. Further stretching in the x- direction was performed to create a uni-axial wrinkled surface, and an ellipse-type mask was attached to the pre-stretched film. The elliptical shape of the mask was necessary to create a circular-type actuator after the film releasing process. Next, carbon particles were brushed along the mask on both sides of the film. After the actuator electrode was coated, the pre-stretched film was released in the x-direction and y-direction. Circular electrodes were formed when the released film reached the initial pre-stretched value.

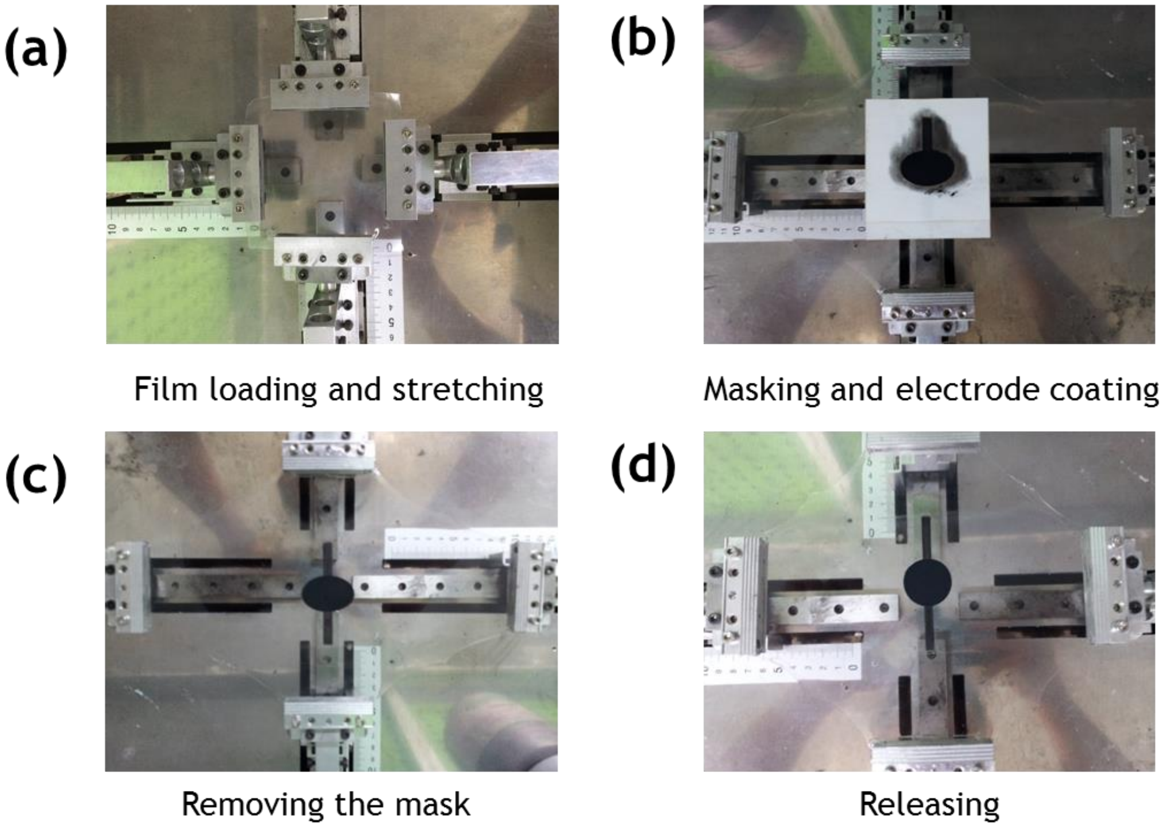

Figure S1. Fabrication steps of wrinkled dielectric elastomer actuator.

Supplementary Note 2. Variation of wrinkle structure with mechanical tensile strain

The variation of electrical resistance and surface morphology were also investigated to verify the operational principle of the directional actuation of the WDEAs as a function of various levels of mechanical strain in **Figure S2**. Image (a) shows a sample under 0 % strain in the x-direction, image (b) is a sample under 20 % tensile strain along the x-direction, and image (c) shows a sample under 20 % tensile strain along the y-direction. The electrical resistance of the wrinkled electrodes was measured along the x-direction, and the results are plotted as a function of the applied tensile strain in the y(■)-direction, and x(■)-direction. In all cases, the arrows indicate the direction of the applied tensile strain.

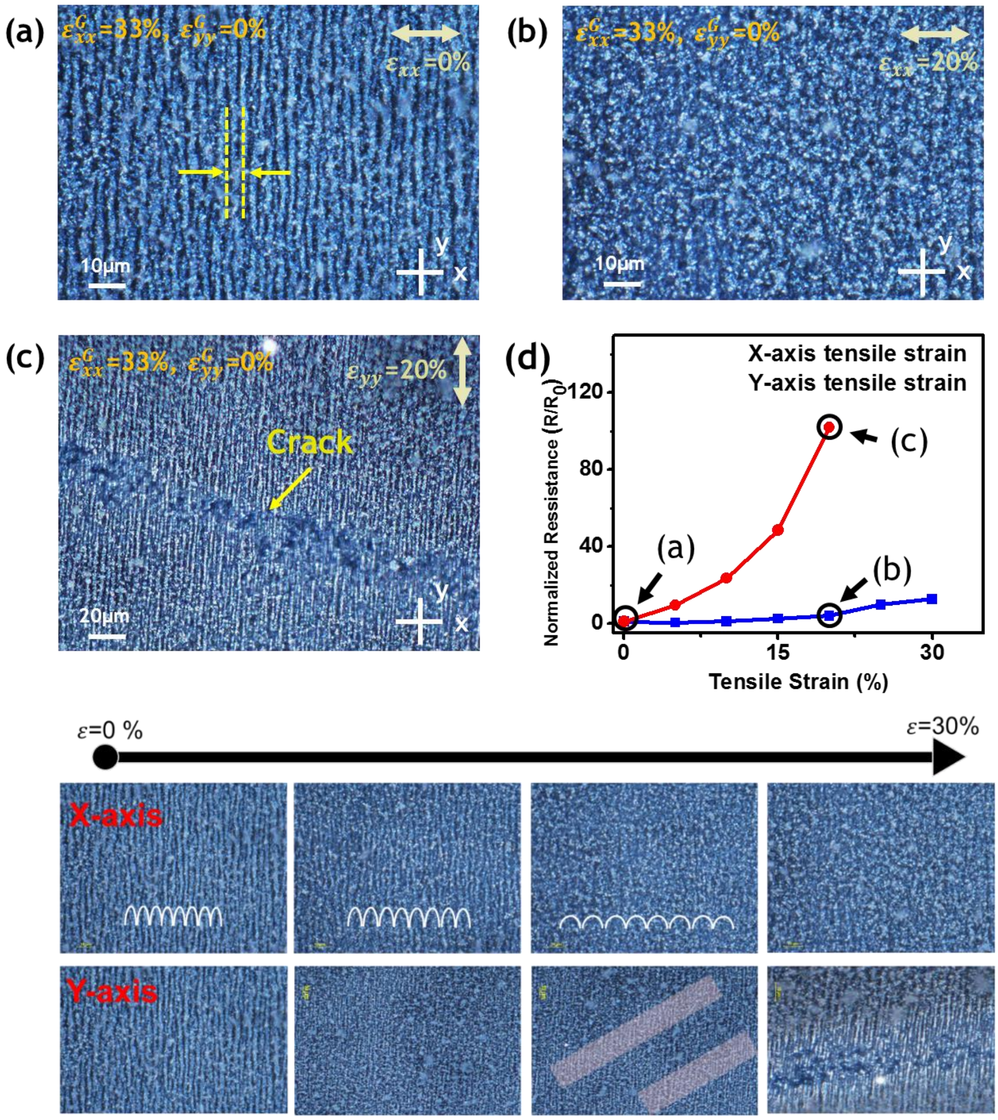

Figure S2. Morphology changes in the electrode surface with mechanical tensile strain.

Supplementary Note 3. Water contact Angle measurements

The contact angle of water droplets on the hydrophobic electrode surfaces was measured using a SEO Phoenix contact angle system (Surface Electro Optics, Korea) in an air environment at room temperature. The volume of the deionized water droplets was ~4 μL, and they were generated by the automatic dispenser of the contact angle system. Using a charge-coupled device (CCD) camera, we took images of the water droplets. For each sample, the water contact angle was measured at five different positions. Measurements were peformed three times at the same position, and the average and deviation values are reported in **Figure S3**.

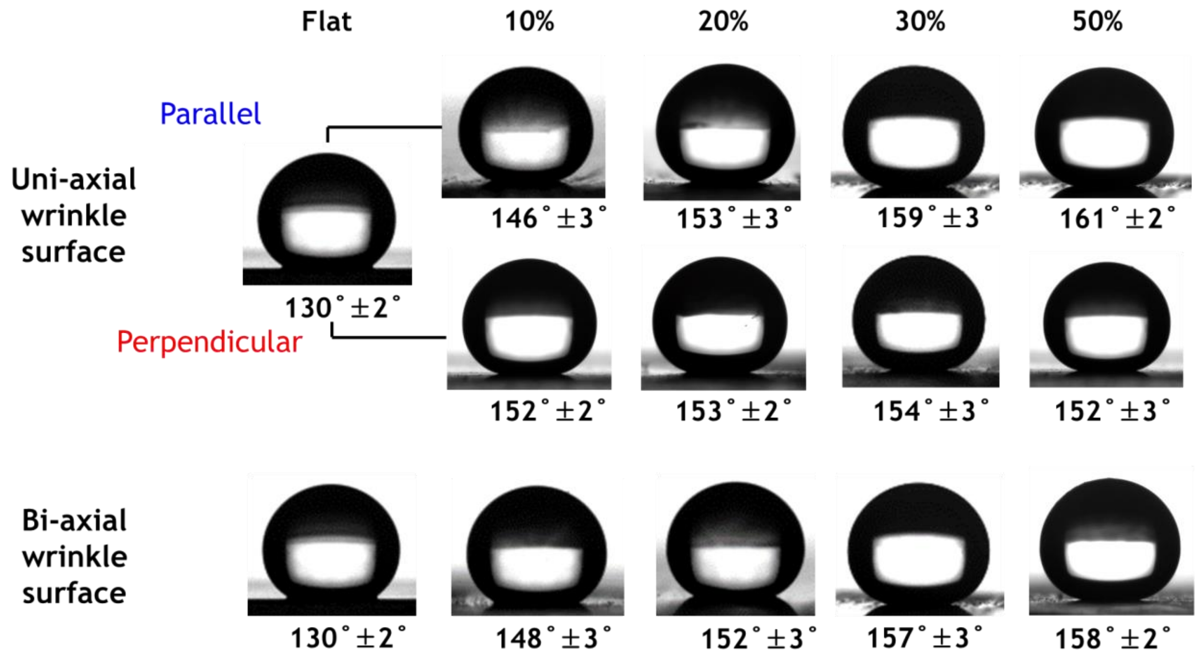

Figure S3. Variations of water droplets as a function of compressive strain.

Supplementary Note 4. Theoretical analysis of anisotropic wetting

It is well known that the end point of the liquid droplet is pinned at the peak points, which are marked as red circles or yellow rectangles in **Figure S4a**. If a height difference exists between the pinning points shown by the red circles and yellow rectangle in **Figure S4a**, the energy barrier between the pinning points is maximized because of the additional liquid-air surface (red dotted in **Figure S4a**). Therefore, the liquid tip will be pinned at locations with large height difference. Also, because the pinning point would not change even though the compressive strain is changed, the base line length of the droplet decreases as the compressive strain increases (as shown in **Figure S4b**), as follows:

$$L = (1 - \epsilon^G)L_0.$$

In the above expression, L refers to the base line length of the droplet, ϵ^G refers to the compressive strain, and L_0 refers to the length between the pinning points of the both ends when $\epsilon^G = 0$. Combining the relation with the constant volume ($V = \frac{L^2}{4\sin^2\theta'}(\theta' - sin\theta' cos\theta')$) of the liquid droplet, the contact angle (θ') for the specific ϵ^G can be obtained by solving a implicit equation,

$$\frac{(\theta' - sin\theta' cos\theta')}{\sin^2\theta'} = \left(\frac{1 - \epsilon^G_{ref}}{1 - \epsilon^G}\right)^2 \frac{\theta'_{ref} - sin\theta'_{ref} cos\theta'_{ref}}{\sin^2\theta'_{ref}}.$$

ϵ^G_{ref} and θ'_{ref} are the reference compressive strain and the contact angle of the surface. In our calculations we used the compressive strain 0.1 and the contact angles at the strain as the reference values.

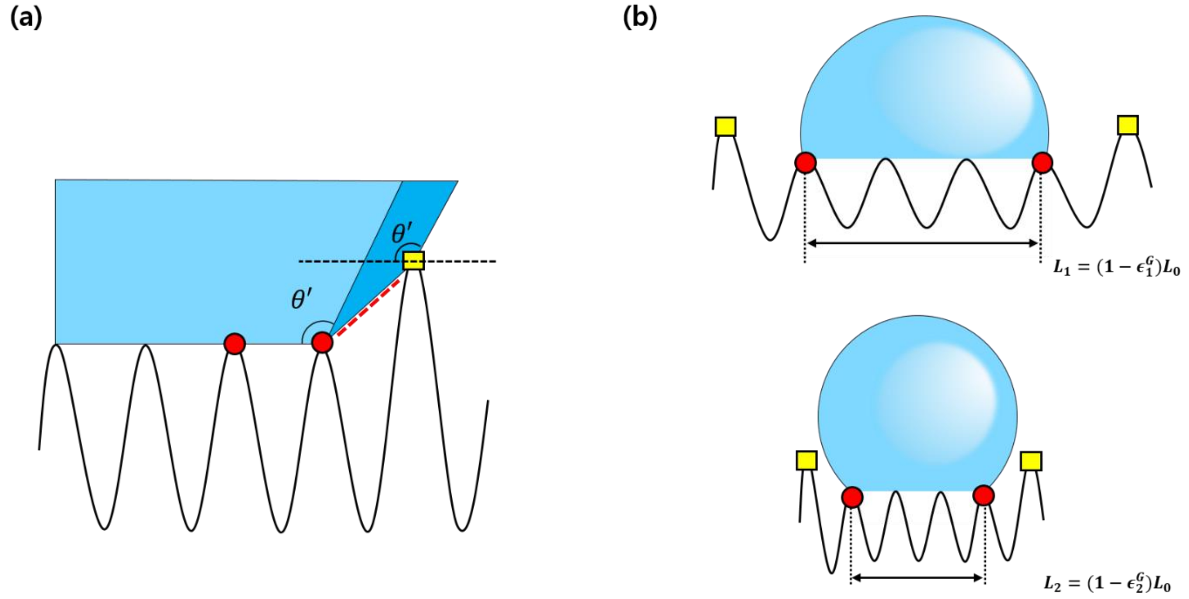

Figure S4. Pinning mechanism: (a) Possible pinning points on the wrinkled surface. The pinning point with a dramatic height difference (yellow rectangular) has a higher energy barrier than the normal pinning points (red circle). (b) Shape of the liquid droplet on the wrinkled surface for various compression strains (ϵ^G). Since the pinning point is preserved even though the compression strain is changed, the base line length of the droplet decreases as the compression strain is increased and consequently, the contact angle increases.

Supplementary Note 5. Measurement setup of water contact angle under activation of elastomer actuator

The experimental set-up used to observe the morphological changes in the elastomer actuator is shown in **Figure S5**. We placed the wrinkled elastomer actuator on a mount. The actuator electrodes were connected by copper tape. After a water droplet was dispensed from a syringe, the syringe was separated from the measurement setup because the syringe needle could influence the water drop. The CCD camera was used to capture the contact angle of each water droplet on the actuator surface. The input voltage range used for activating the actuator ranged from 0 kV to 6 kV.

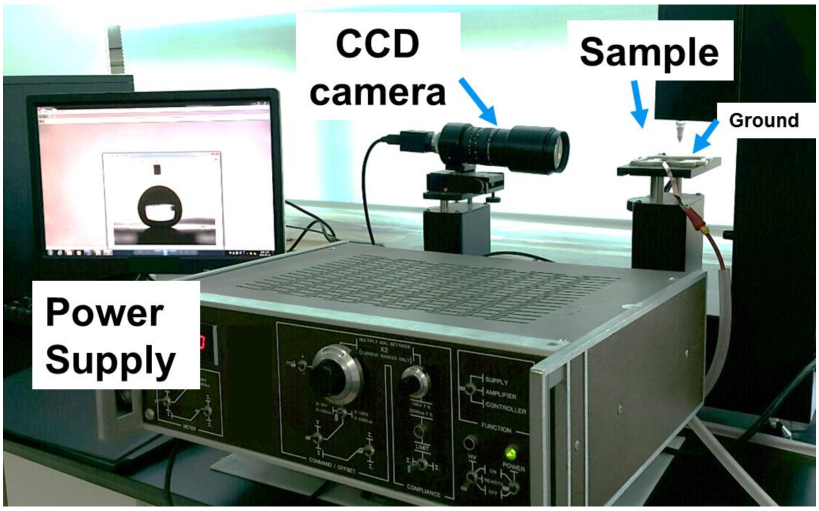

Figure S5. Photograph of water contact angle test set-up to confirm the change in morphology and roughness with WDEA actuation.

Supplementary Note 6. Macroscopic observation of anisotropic actuator under various conditions

The experimental conditions and the actuation results of the elastomer actuators are shown in Table S1. Three types of elastomer actuators were used in this experiment. In **Table S1**, W-01 did not have a wrinkled surface, and was used as a reference sample. The others had different levels of wrinkle conditions. Also, we recorded the maximum actuation strain value before breakdown voltage.

Table S1. Anisotropic behavior of WDEAs under different conditions.

Specimen	y-axis Pre-strain [%]	x-axis Prestrain [%]	x-axis Relaxation [%]	Compressive Strain, ε_G [%]	x-axial Wrinkle Pattern	Max Areal Strain, s_{A_max} [%]	Max x-axis Strain, s_{x_max} [%]	Max y-axis Strain, s_{y_max} [%]	Anisotropy (s_{x_max}/s_{y_max})
**W-01 (Reference)**	100	100	100	0	X	70.66	31.63	30.48	1.04
**W-02**	100	150	100	20	O	82.14	35.82	30.91	1.16
**W-03**	100	200	100	33.3	O	90.25	40.95	31.21	1.31
